# Immune Evasion as the Main Challenge for Immunotherapy of Cancer

**DOI:** 10.3390/cancers14153622

**Published:** 2022-07-26

**Authors:** Radoslaw Zagozdzon, Magdalena Winiarska, Malgorzata Firczuk

**Affiliations:** 1 Department of Clinical Immunology, Medical University of Warsaw, Nowogrodzka 59 Street, 02-006 Warsaw, Poland; 2 Department of Immunology, Medical University of Warsaw, Nielubowicza 5 Street, 02-097 Warsaw, Poland; mwiniarska@wum.edu.pl (M.W.); mfirczuk@wum.edu.pl (M.F.)

Immune evasion is currently considered one of the most prominent hallmarks of cancer [1]. The immune system has evolved to perform two main functions: (1) elimination of the pathogenic danger and (2) tolerance toward self-tissues or symbiotic organisms. Additionally, the immune system can aid damaged tissue in the healing process. Under favorable conditions, malignant transformation is recognized as foreign and dangerous, and the immune system initiates the elimination attempt. This concept is referred to as immune surveillance [2]. If the immune surveillance succeeds, cancer can be eliminated before it becomes clinically detectable. However, in case of incomplete success, the selective pressure from the immune system initiates Darwinian-type microevolution [3]. In this process, called immunoediting, cancer evolves towards evading the destructive potential of the immune system, while taking advantage of the self-tolerance mechanisms or the pro-regenerative capabilities of the immune cells. There are multiple paths that cancer can follow during the escape from immune destruction. One of them occurs in so called “cold tumors”, when tumor microenvironment is devoid of effector cell infiltration capable of eliminating the cancer. An opposite situation is the “hot tumors”, when infiltrating tumor T cells are actively disarmed by a range of immunoregulatory mechanisms. Here, the main role in the process of immune evasion is attributed to suppressive subtypes of immune cells along with the effects elicited by metabolic and immune checkpoints. Quite often a mixture of evasion mechanisms occurs, synergizing towards the immune escape and progression of cancer. Understanding in detail the crosstalk between the tumor and immune system is one of the main challenges for oncological research in order to design new and more efficient immunotherapies [4]. It becomes obvious that successful anticancer treatment requires a holistic view on the subject of immune evasion.

This special issue is dedicated to the elucidation of a range of mechanisms responsible for tumor immune evasion, as well as potential ways of battling these mechanisms. Reviews and research papers gathered in the current special issue describe several crucial aspects of the immune escape. Specifically, reviews by Pastorczak et al. [5] and Swatler et al. [6] provide overviews on the immune evasion mechanisms in hematological malignancies—acute lymphoblastic and myeloid leukemias, respectively. In turn, the works by Retecki et al. [7] and Anichini et al. [8] summarize current knowledge on the immune landscape of breast cancer and non-small cell lung cancer, respectively, as examples of solid tumors. Six of the papers focus on the role of specific cell types in tumor immune evasion. Among these, the research paper by Menzner et al. [9] reported a novel axis between hydrogen peroxide and low-density lipoproteins-mediated axis in immunosuppressive abilities of monocyte-derived dendritic cells. The review by Demkow [10] underlined the multifaceted crosstalk between cancer and neutrophil extracellular traps during tumor progression. Macrophages, as main players in tumor-promoting inflammation and the dialogue between the immune system and cancer, were the subject of a comprehensive review by Cendrowicz at al. [11]. Additionally, the most promising new strategies for targeting the cell surface molecules present on tumor-associated macrophages to improve antibody-based therapies were presented in the paper by Hussain at al. [12]. An attractive point of view was presented by Grzywa et al. [13] in a review describing the potential role of dysregulation of erythroid progenitor cells, among other immunosuppressive cell types, in the interactions between the hematopoietic/immune systems and developing cancer. The review by Domagala et al. [14] described the effects of tumor microenvironment-related metabolic factors on the anticancer effectiveness of natural killer cells. Finally, the review by Zhylko et al. [15] presented strategies for overcoming the immune escape mechanisms for the successful application of novel anticancer immunotherapies utilizing chimeric antigen receptor-bearing T cells. On the molecular level, a research article by Jancewicz et al. [16] and a review paper by Glorieux et al. [17] focus on highlighting specific associations between PD-L1-mediated immune checkpoint functions in cancer in correspondence to SWI/SNF complex deregulation and redox signaling, respectively. Molecular insights were also provided in the review by Tanaka and Siemann [18] regarding the role of Gas6/Axl-mediated axis in the tumor immune evasion mechanisms.

Altogether, the set of articles in the current special issue presents an extraordinary variety of events that may occur when developing cancer escapes immune surveillance and shapes the microenvironment to support tumor development and progression. It is becoming apparent that for the holistic understanding of these multilayer relations between cancer and the immune system, the generation of novel, more precise preclinical models is needed in addition to the existing diagnostic and research methodologies. Among others, the hope lies in combining recent advances of the 3D-bioprinting systems, as reviewed by Staros at al. in the current issue [19] and other authors [20,21,22], with single-cell- and multi-omic-based assays [23,24] along with the strong support from mathematical and computational oncology tools [25]. Such structures can more precisely recapitulate the specificities of the tumor microenvironment and provide vital information for generating new and successful anticancer immunotherapeutics.
